# Family physician attitudes in managing obesity: a cross-sectional survey study

**DOI:** 10.1186/1756-0500-4-473

**Published:** 2011-11-01

**Authors:** John W Epling, Christopher P Morley, Robert Ploutz-Snyder

**Affiliations:** 1 Department of Family Medicine and Department of Public Health and Preventive Medicine, SUNY Upstate Medical University, Syracuse, NY, USA; 2 Department of Psychiatry, SUNY Upstate Medical University, Syracuse, NY, USA; 3 Universities Space Research Association, Division of Space Life Sciences, Houston, TX, USA

## Abstract

**Background:**

Obesity is epidemic in primary care. While family physicians care for the consequences of obesity, they do not generally feel confident managing obesity itself. We examined the barriers to obesity management in a sample of family physicians in a primary care practice-based research network (PBRN).

**Findings:**

204 family physicians were invited to respond to a survey on physician beliefs about obese patients and causes of obesity. A total of 75 physicians responded to the survey. Responses were factor analyzed using standard techniques. Comments were sorted into ranked themes by the investigators. The results show systemic barriers to obesity management. Seven general factors were identified, with some discrepancy seen in the role of "psychobehavioral causation" between rural and non-rural physicians. Themes derived from the comments reflected frustration with the resources and structure of current primary care systems to be able to deal with obesity.

**Conclusions:**

Our pilot survey suggests that differences in beliefs regarding the causes of obesity may exist between rural and non-rural physicians. Further research in larger, more diverse samples is necessary to further illuminate practice differences. More comprehensive approaches to obesity management, like the Chronic Care Model, are suggested by these results.

## Background

Over the last 20 years obesity has risen to epidemic proportions, leading to the latest recommendation by the US Preventive Services Task Force that adults be screened for overweight and obesity (via calculation of the Body Mass Index) and that identified cases be followed up with weight loss counseling and behavioral modifications [1]. Implementation of these recommendations in primary care settings, however, has been difficult. For example, Ma and colleagues found that physicians who had participated in the National Ambulatory Medical Care Survey had only identified 29% of adult obese patients during office visits [2]. Similarly, Yaemsiri and colleagues (2010) have reported that 74% of overweight and 29% of obese individuals have not received a formal diagnosis of overweight or obesity, using data from the 2003 to 2008 National Health and Nutrition Examination Survey. They point out that primary care providers "have unused opportunities to motivate their patients to control and possibly lose weight by correcting weight perceptions and offering counseling on healthy weight loss strategies" [3]. Many barriers that primary care physicians face when implementing the guidelines for the prevention and treatment of obesity have been described in the literature for both adults and children, in a variety of settings, and across international borders. These include negative characterization of obese patients, lack of support services, failure to implement office systems to screen for obesity, and lack of training and self-efficacy in counseling for obesity and general lifestyle management [4-21].

To examine this problem on a local level as a needs assessment for further work in this area, we initiated an attitudinal survey of primary care physicians located in Central New York in the Spring and Summer of 2007, asking about perceived causes of obesity, comfort with and accommodations for obese patients, and barriers to implementation of interventions. The survey was administered as part of an inaugural recruitment effort for a regional practice-based research network (PBRN) centered in Syracuse, New York, and included rural, suburban, small city, and mixed practices.

## Methods

### Study Design

We used a cross-sectional survey instrument, adapted from a set of previously published survey questions [8], to assess primary care physicians' attitudes toward obesity and their perceptions of difficulties in the management of obesity. We also gathered basic demographic information (age, sex, specialty, years in practice, setting of practice), for a total of 31 Likert-scaled (1-5) attitudinal and opinion questions, and 6 demographic questions with qualifiers. We additionally included open-ended questions to collect qualitative data on specific needs perceived by the respondents. A cover letter explained the nature of the survey, informed the recipients about the development of a PBRN and invited them to participate in both the network and the survey. The instrument was distributed via both a mail-delivered paper form, as well as via an online version through e-mail invitation. A network recruitment form accompanied the survey instrument in both cases, and respondents were made aware that their participation in either the survey or the PBRN were unlinked and completely voluntary. Only one email & paper mail reminder was sent, approximately three weeks after the first survey invitation was distributed. This decision was made in order to avoid creating ill-will toward the new network by sending unwanted solicitations. This study was deemed exempt from review by the Institutional Review Board for the Protection of Human Subjects at SUNY-Upstate Medical University. The invitation to participate in the study was included in the email and letter, including risks of participation and the voluntary nature of the study, and that submitting a survey response was equivalent to a signed affirmation of informed consent.

### Sample Population

Participants were recruited from existing educational networks of primary care physicians affiliated with SUNY-Upstate Medical University's Department of Family Medicine. These networks comprise primary care (mainly family physician) practices in the urban, suburban and rural areas in Central and Northern New York.

### Analysis

All statistical analyses were conducted using the Statistical Package for the Social Sciences (SPSS), v. 15.0.1. Factor Analysis of the 31 Likert-scaled items was conducted using Principal Axis Factoring, with varimax rotation and pairwise elimination, and factor scores were derived via regression methods that utilize item scores weighted by eigenvalues. Group differences on factor scores for Rural vs. non-Rural, Sex, and Race/Ethnicity were analyzed by Student's T-test, and Pearson's correlations were calculated for all items to analyze between-variable association. Analysis of Covariance was used to compare Rural vs. Non-Rural Factor Scores with Experience Years as a covariate, and using participants' self-reported Rural/non status, since those data were available for all participants.

The qualitative data were open-coded by 2 investigators (JWE & CPM), and themes were developed from these codes. The themes were then ranked by frequency of mention by survey respondents.

## Results

Approximately 204 survey invitations were sent to regional primary care physicians via both an e-mail invitation to participate in an online version of the survey, as well as a paper copy and letter invitation, with 75 responding. We were able to obtain usable data for 67 respondents with completed surveys, and 5 partially completed surveys. Respondents were all family physicians, with the exception of a single internal medicine specialist. Reported practice settings were rural (N = 35) or suburban (N = 25), with 5 urban and 2 "other" settings specified. The largest urban setting in Central New York is the city of Syracuse, itself relatively small and surrounded by suburban and rural areas. For the purposes of analysis, suburban, urban, and other practice settings were reduced to a single "non-rural" category, due to the level of similarity between urban and suburban practices in our region. No significant differences were found between these settings on any measure.

The mean (SD) number of years in practice for the sample was 20.16 (7.19), and the mean age was 50.72 (8.31). Slightly over 73% of the sample was male (male n = 49, female n = 18), 95% White (64/67) and 99% non-Hispanic (66/67). Self-reported rural physicians were also significantly older than non-rural physicians (rural mean age: 54.80 years, SD = 6.98, non-rural mean age: 46.12 years, SD = 7.27; p < .000), and had more years in practice (rural: 23.97 years, SD = 7.23, non-rural: 16.00 years, SD = 6.46; t = -4.745, p < .000). Of the 75 total responses, 41 were returned online, and 34 were returned on paper. There was no significant difference between the two groups in terms of age (online responder average age was 51.3, vs. 49.8 for mail responders). The gender distribution between response types mirrored the overall gender breakdown, with 30 of 41 online responses coming from males (73.17%), whereas 20 of 34 paper responses came from males (58.82%), a difference which was statistically significant (χ^2 ^= 4.836, p = .028).

The 31 Likert-scaled items and responses to each are summarized in Table 1 and are organized by frequency of response. Principal axis factor analysis (Varimax rotation, Kaiser normalization) of these responses, with exclusion of single-item factors and items which did not appear in any factor, revealed 7 underlying constructs. We elected to describe the first factor grouping as a "medical causation" belief structure (encompassing endocrine, metabolic, and genetic factors, along with a tendency to reject the idea that obesity was under the patient's control); "motivational causation," which appears to group those who agree that physical inactivity, overeating, a lack of will-power, and restaurant eating were fundamental to obesity; items which described comfort with and behavior towards obese patients; interestingly, a separate factor grouping restaurant eating with psychological problems, repeated dieting and weight cycling emerged, separate and apart from the "motivational causation" factor, indicating an apparent split between those who hold a more pejorative view of obese patients and those who view obesity as a behavioral issue - which we can call a "psycho-behavioral causation" factor; and additional factors that grouped respondents according to "aggressive physician role," "medication usage," "physician nihilism," and "physician dissympathy." These factors and specific components of each are summarized in Table 2.

**Table 1:** Summary of 31 Likert-scale attitudinal and opinion questions (Adapted from Foster^6^)

Prompts	Mean	SD
I believe it's necessary to educate obese patients on the health risks of obesity	4.81	0.52
Obesity is a chronic disease	4.86	0.43
I make accommodations for obese patients	3.79	0.92
Obesity is associated with serious medical conditions	4.93	0.32
Physicians should be role models by maintaining a normal weight	4.21	0.92
A 10% reduction in body weight is sufficient to significantly improve obesity-related health complications	3.91	0.81
I would spend more time working on weight management issues if my time was reimbursed appropriately	4.01	1.08
I feel competent in prescribing weight loss programs for obese patients	3.71	1.02
Most obese patients are well aware of the health risks of obesity	3.41	1.08
Medications to treat obesity should be limited to short-term (3 months) use	3	1.23
Most obese patients could reach a normal weight (for height) if they were motivated to do so	2.53	1.09
Most obese patients will not lose a significant amount of weight	3.63	1.08
I have negative reactions towards the appearance of obese patients	2.8	1.15
'If a patient meets the appropriate criteria for obesity surgery, I would recommend an evaluation by a surgeon'	4.17	0.85
Medications to treat obesity should be used chronically	2.64	1.24
I am usually successful in helping obese patients lose weight	2.5	1.00
'For most obese patients, long-term maintenance of weight loss is impossible'	2.79	1.05
It is acceptable to use "scare tactics" to obtain compliance of the obese patient	2.59	1.16
I feel uncomfortable when examining an obese patient	1.77	0.92
It is difficult for me to feel empathy for an obese patient	1.81	0.95
**The factors below are important causes of obesity**:		
Physical inactivity	4.62	0.64
Overeating	4.67	0.48
High-fat diet	4.17	0.91
Genetic factors	4.47	0.63
Poor nutritional knowledge	4.23	0.91
Psychological problems	4.12	0.81
Repeated dieting (weight cycling)	3.85	0.89
Restaurant eating	3.85	0.95
Lack of willpower	3.71	0.92
Metabolic defect	3.49	0.99
Endocrine disorder	2.96	1.04

Responses were scaled 1-5, with 5 indicating "Strongly Agree" and 1 indicating "Strongly Disagree." Response N for each question varied between 66 and 70 responses.

**Table 2:** Factor analysis by principal axis factoring w/Varimax rotation with Kaiser normalization, revealed 7 factors, interpreted below

Factor Name	Individual Items	Rotated Item Load	Comment
	*Metabolic defect*	0.79	
	*Endocrine disorder*	0.77	
**Factor 1:** **Medical Causation**	*Most obese patients could reach a normal weight (for height) if they were motivated to do so*	-0.55	Attribution of obesity to metabolic, endocrine & genetic causes is associated with accommodation for obese patients, and inversely associated with ability of patients to control obesity
	*Genetic factors*	0.53	
	*I make accommodations for obese patients*	0.43	
		
	*Physical inactivity*	0.78	
**Factor 2:** **Motivational Causation**	*Overeating*	0.61	Factors related to a more pejorative attitude toward obese patients are distinct from both medical cause as well as apparently psychopathological causes
	*Lack of willpower*	0.54	
	*Restaurant eating*	0.43	
		
	*Physicians should be role models by maintaining a normal weight*	0.81	
	*It is acceptable to use "scare tactics" to obtain compliance of the obese patient*	0.56	
**Factor 3:** **Aggressive Physician Role**	*I feel competent in prescribing weight loss programs for obese patients*	0.54	Use of medication chronically, scare tactics, physician role-modeling & accommodation all grouped together, indicating a more aggressive approach to obesity intervention
	*I make accommodations for obese patients*	0.47	
	*Medications to treat obesity should be used chronically*	0.44	
	*Physicians should be role models by maintaining a normal weight*	0.81	
		
	*Repeated dieting (weight cycling)*	0.83	
**Factor 4:** **Psychobehavioral Causation**	*Restaurant eating*	0.71	Attribution of obesity to psychological problems and weight cycling may be tied together under eating disorders, along with restaurant eating
	*Poor nutritional knowledge*	0.54	
		
**Factor 5:** **Dissympathy**	*I have negative reactions towards the appearance of obese patients*	0.85	Reactions toward obese appearance & difficulty w/empathy are associated; positively correlated w/age & years in practice
	*It is difficult for me to feel empathy for an obese patient*	0.82	
		
**Factor 6:** **Medication Usage**	*Medications to treat obesity should be limited to short-term (3 months) use*	-0.87	Physicians who support long-term or chronic use of medication to control obesity tend to not agree with limits on term of use and vice versa
	*Medications to treat obesity should be used chronically*	0.68	
		
	*Most obese patients are well aware of the health risks of obesity*	0.66	
**Factor 7:** **Physician Nihilism**	*Most obese patients will not lose a significant amount of weight*	0.62	The assumptions that patients are aware of health risks, and yet won't lose weight is associated with an assumption about low likelihood of success in helping these patients lose weight
	*I am usually successful in helping obese patients lose weight*	-0.59	

Single-item factors, and items which did not appear in any factor, where excluded.

Correlation analysis between family physician characteristics and factor scores for each of the 7 identified factors described above revealed significant inverse relationships between the behavioral factor and age (r = -.320, p = .017) as well as years in practice (r = -.344, p = .010). The relationship between the physician dissympathy factor and both age (r = .248, p = .067) and years in practice (r = .253, p = .062) approached traditional levels of statistical significance. We also found a significant difference in age (p <. 01) and years of experience (p < .01) between our self-reported rural vs. non-rural physicians. In our sample, the rural family physicians were significantly older than non-rural family physicians (means (SD) = 54.8(6.98) and 46.12(7.27), respectively) and had significantly more years of experience (means {SD} = 23.978 {7.23} and 16.00 {6.46}, respectively).

We performed analysis of covariance (ANCOVA) to compare the seven factor scores between self--reported rural and non-rural physicians using years of experience as a covariate. Our analyses revealed significant differences between self-disclosed rural physicians and non-rural family physicians on the psychobehavioral factor score. Specifically, self-reported non-rural family physicians tended to endorse psychobehavioral contributors to obesity (repeated dieting, psychological problems, repeated restaurant eating) more than rural physicians (p = .013), even after factoring in years of experience. None of the other factor scores were significantly different for self-reported rural vs. non-rural family physicians.

The qualitative themes uncovered from the comments (Table 3) reveal a significant frustration with the current medical system's ability to address a complex problem such as obesity. Participants decried a lack of reimbursement and time to manage obesity, felt generally unprepared to do so (lack of adequate training, guidelines, resources, referral options), and felt that many factors were beyond their control (the environment, futility/determinism, and the patient's role in the problem).

**Table 3:** Themes from open-ended survey comments, ranked by frequency of mention

Theme	Frequency of mention	Comments
Better Interventions Needed to Treat Obesity	40	Poor access to bariatric surgery, few other manageable alternatives
Poor Reimbursement for Management of Obesity	35	Counseling, preventive services often not covered in primary care
Obesogenic Environment	30	The "built environment" encourages obesity
Inadequate Time for Management of Obesity	28	Too many other distractions
Better Access and/or Reimbursement to Fitness/Coaches/Dieticians Needed	25	Better integration of non-physician support needed
The Patient is to Blame for Their Obesity	16	Overeating, restaurant eating, laziness
Better Referral Systems are Needed to Manage Obesity	14	Few pathways to bariatrics, dieticians, etc.
Screening is Non-Issue	13	Screening already performed well-enough - treatment is the difficulty
Futility of Attempts to Manage Obesity	8	Relates to both patient blame and to lack of time and referral pathways
Better Guidelines Needed for Obesity Management	6	Existing guidelines not realistic for practice
Better Patient Education Materials Needed for Obesity Management	6	Including culturally appropriate education materials
Group Visits Needed to Treat Obesity	4	Suggested; no clear indication whether these were implemented.
Better Screening Tools Needed to Identify Obesity	4	Related to general dissatisfaction with existing screening & management tools
Better Goal Setting Needed to Manage Obesity	2	Related to guidelines & educational materials
Better Physician Training Needed in Obesity Management	2	Similar to need for better guidelines
Discomfort of Physicians with Subject of Obesity	2	Not wanting to cross a barrier with patient.
General difficulty with topic	2	It is difficult to convince patients of the problem and to get to new
Staffing	2	Staff assistance is needed
Bariatric Surgery Endorsement	1	"surgery works"
Fatalism	1	"childhood determines risk"
Difficulty in Management	1	"paperwork"

## Discussion

This study describes a general sense of frustration among family physicians in managing obesity - stemming from a lack of adequate compensation for treatment efforts, a general lack of self-efficacy, and a complex set of factors that seem to be perceived as beyond the physician's control. These findings parallel the results of the study by Foster, et al. [8], from whom the survey instrument for this study was adapted.

An interesting finding for this study is the detection of a difference in physician opinions and attitudes regarding the causes of obesity, between self-defined rural and non-rural physicians in our sample. This difference accounted for approximately 19% of variance in the attribution of psycho-behavioral factors to obesity, without the inclusion of any other independent variables. Our sample was admittedly small, limited in geographic scope to one section of New York State, and essentially limited to one medical specialty (Family Medicine), so the generalizability of this specific finding requires verification in other larger and more diverse samples. However, a corollary to the split on this particular factor along rural/non-rural lines is that such a finding may indicate a substantive difference in belief sets between those who practice in rural settings, vs. those who practice in other, non-rural settings, as Rabinowitz and others have maintained [22].

The fact that rural physicians in our sample tended to be older - and consequently have more years in practice - may be tied in some way to the opinion split, although we found no explicit statistical effect indicating this is the case. Nevertheless, it is possible that a more sensitive analysis in a more diverse sample might indicate a stronger effect for age or practice-length differences. Additionally, in our sample, these two variables were so closely correlated as to be nearly indistinguishable statistically. It is not unreasonable to suspect that, in a sample where age and years in practice were not so closely correlated, an age, practice-length, or even a generational effect might be demonstrable. Samples containing a large number of physicians who entered the profession later in life, for example, might indicate generational effects. Regardless, such an analysis was not possible with our current data set. When viewed alongside the fact that we found nearly significant indications of lower empathy and greater discomfort in older and more experienced physicians, it is clear that additional study of demographic effects upon physician beliefs and practices may be warranted.

A potential weakness of the conclusion based on rurality is the limitations of self-report data in defining rural practice. In the case of the population we surveyed here, as well as the particular practices surveyed, we highly suspect that the self-reports of practice setting given by our respondents often reflected the population primarily seen by the practice, as opposed to the physical location of the practice. However, while self-report may be an apparently unreliable source of information about the physical location of the practice, we suspect it is still useful for delineating physicians who view themselves as treating rural patients, as opposed to those who do not perceive themselves to be doing much or any such practice.

There are a number of limits to the generalizability of this study. The sample was relatively small and homogenous, representing essentially one medical specialty in one region. Additionally, the differences between practicing in a small city, a suburb of that city, or the outlying rural areas, may be somewhat small, compared with a national sample that including both very large, major urban centers as well as very remote rural regions. However, this apparent weakness may also serve to further emphasize differences we did find related to self-reported practice setting. The collected sample was over-representative of white/Caucasian physicians, and slightly over-representative of males compared to the membership of the American Academy of Family Physicians (73% male in our sample vs. 63% male AAFP membership) as of 2009 [23]. The survey also did not ask physicians about their own perceived responsibility in managing their patients' weight. A number of studies have identified questions on the part of primary care providers about the extent of physician responsibility for patient weight loss, and this seems to be an underlying factor at play in the current analysis [13,18,24,25]. Finally, the study did not ask respondents to comment upon their use of or referral to commercial weight loss products and programs. As this industry continues to grow, the use, implementation and acceptance of such products, services and systems warrants additional study.

The current study indicates that, regardless of setting, primary care physicians appear to need more precise guidelines and better tools for screening and management of obesity, more referral options, better reimbursement for obesity-related services, improved coordination with non-physician providers, and reimbursement of patients for activities such as dietician consultation and fitness activities. Additionally, issues such as culturally appropriate care and discomfort with the subject of obesity may be partially ameliorated with training. For example, better word choice may improve the discussion when physicians are uncomfortable broaching the subject of obesity [26].

More globally, the barriers identified align with those that would be addressed by the adaptation of the principles of the Chronic Care Model and the Patient-Centered Medical Home, both by providers and insurers [27-31]. The Chronic Care Model, originally championed by Wagner [32,33], involves the use of patient education, systems-level disease management protocols, goal setting, and patient self-management. When incorporated into a broader framework of patient-centered practice, CCM-based management of obesity (and reimbursement of the primary care team for it) would likely be optimal in addressing the recommendations listed above.

## Conclusions

This study on the barriers to obesity management in primary care reveals a number of areas requiring intervention as well as further study, but was a pilot study, made possible with seed funding for the creation of a new PBRN. With only 75 responses from a limited geographic region, we have reinforced previous study in the area of barriers, and we believe our results offer preliminary evidence of attitudinal differences toward obesity and obese patients between those physicians who self-identify as rural, vs. those who do not. More research in this area is clearly needed. While greater education of physicians in the topic of obesity causation and management is needed, a more systemic approach to improving resources, patient self-management and the healthcare system's response would more completely address the barriers found in our study.

## Competing interests

The authors declare that they have no competing interests.

## Authors' contributions

JWE designed and implemented the study and co-authored the paper. CPM implemented the study, provided statistical consultation and co-authored the paper. RPS assisted with the technological implementation of the survey, provided statistical consultation and reviewed and edited the paper. All authors reviewed and approved the final draft of the paper.
